# Effect of microRNA-21 on the proliferation of human degenerated nucleus pulposus by targeting programmed cell death 4

**DOI:** 10.1590/1414-431X20155020

**Published:** 2016-05-24

**Authors:** B. Chen, S.G. Huang, L. Ju, M. Li, F.F. Nie, Y. Zhang, Y.H. Zhang, X. Chen, F. Gao

**Affiliations:** 1 Department of Orthopedics, Linyi Second People's Hospital, Linyi, China; 2 Department of General Surgery, Affiliated Hospital of Taishan Medical University, Taian, China

**Keywords:** Intervertebral disc degeneration, MicroRNA-21, Human nucleus pulposus cells, Matrix metalloproteinase-2, Matrix metalloproteinase-9, Target gene

## Abstract

This study aims to explore the effect of microRNA-21 (miR-21) on the proliferation of human degenerated nucleus pulposus (NP) by targeting programmed cell death 4 (PDCD4) tumor suppressor. NP tissues were collected from 20 intervertebral disc degeneration (IDD) patients, and from 5 patients with traumatic spine fracture. MiR-21 expressions were tested. NP cells from IDD patients were collected and divided into blank control group, negative control group (transfected with miR-21 negative sequences), miR-21 inhibitor group (transfected with miR-21 inhibitors), miR-21 mimics group (transfected with miR-21 mimics) and PDCD4 siRNA group (transfected with PDCD4 siRNAs). Cell growth was estimated by Cell Counting Kit-8; PDCD4, MMP-2,MMP-9 mRNA expressions were evaluated by qRT-PCR; PDCD4, c-Jun and p-c-Jun expressions were tested using western blot. In IDD patients, the expressions of miR-21 and PDCD4 mRNA were respectively elevated and decreased (both P<0.05). The miR-21 expressions were positively correlated with Pfirrmann grades, but negatively correlated with PDCD4 mRNA (both P<0.001). In miR-21 inhibitor group, cell growth, MMP-2 and MMP-9 mRNA expressions, and p-c-Jun protein expressions were significantly lower, while PDCD4 mRNA and protein expressions were higher than the other groups (all P<0.05). These expressions in the PDCD4 siRNA and miR-21 mimics groups was inverted compared to that in the miR-21 inhibitor group (all P<0.05). MiR-21 could promote the proliferation of human degenerated NP cells by targeting PDCD4, increasing phosphorylation of c-Jun protein, and activating AP-1-dependent transcription of MMPs, indicating that miR-21 may be a crucial biomarker in the pathogenesis of IDD.

## Introduction

Chronic low back pain affects approximately 70% of people at some point in their lives, with around 10% being chronically disabled (1). The causes of low back pain are multifactorial, but 40% of cases involve intervertebral disc degeneration (IDD) (2). Moreover, IDD is also recognized as a major pathological cause of spinal degenerative diseases and acute lumbar radiculopathy, imposing burden on the health care system, and in social and economic development worldwide (3–5). IDD patients always present characteristics such as reductions in nucleus pulposus (NP), proteoglycans, aggrecan and collagen (6,7). IDD is caused by progressive disk degradation and concomitant disk adaptation, as disc structures and vertebrae are remodeled in response to physical loading and external injuries (5). Accumulating studies have shown that IDD can be attributed to various pathogeneses, all of which induce massive apoptosis and necrosis of intervertebral disc (IVD) cells and of the extracellular matrix (ECM) (1,8). The basic pathogenic, cellular and molecular mechanisms of IDD, however, are still not fully elucidated.

MicroRNAs (miRs) have drawn increasing attention for their potential regulatory roles in the physiologic and pathological processes of IDD (9). Notably, miRs mediate a number of biological functions by sequence-specific modulation of gene expression at post-transcriptional level, particularly post-transcriptional regulation of protein expression and regulation of mRNA stability (10,11). Consequently, miRs are also seen as new therapeutic targets in many diseases (12,13). In osteoarthritis and rheumatoid arthritis, miRNAs may play an indispensable role in regulating cell development, metabolism, and apoptosis of degenerated cartilage (14,15). Furthermore, miR-21 can promote the apoptosis of dendritic cells, playing a crucial role in the regulation of the body's immune system, inflammation and apoptosis (16,17). Intriguingly, a previous study reported an important functional linkage between miR-21 and tumor suppressor PDCD4, by which the overexpression of miR-21 can contribute to inhibition of PDCD4 and its tumor-suppressive functions (18). However, few researches have investigated how the specific miR-21-PDCD4 association acts in IDD. To our knowledge, the role of miR-21 in IDD, as well as its mechanism, remains unknown.

Matrix metalloproteinase (MMPs) can be grouped into three classes according to the substrates they degrade: collagenases (such as MMP-1 and MMP-13), gelatinases (including MMP-2 and MMP-9) that act on denatured collagens, and stromelysins (such as MMP-3) (19). MMP-2 and MMP-9 were selected in our study as they have been commonly described in association with IDD, and are responsible for matrix degradation (20). The purpose of this study was to explore the effect of miR-21 on the proliferation of human degenerated NP by targeting PDCD4.

## Material and Methods

### Subjects and study design

This study was approved by the Institutional Review Board of the Linyi Second People's Hospital. Written informed consent was obtained from all participants. Ethical approval for this study conformed to the standards of the Declaration of Helsinki (21).

IDD NP tissues were collected from patients undergoing discectomy in the Department of Orthopedics, Linyi Second People's Hospital from June 2013 to June 2014. This study enrolled a total of 20 patients (11 males, 9 females) ranging from 26 to 65 years old, and including L4/5 (n=12) and L5/S1 disk herniations (n=8). Selection criteria were as follows: patients had typically clinical symptoms of lumbar disc herniation; preoperative X-ray showed lesions with narrowing of the intervertebral-disc space and part of compensatory lumbar idiopathic scoliosis; preoperative computed tomography (CT) and magnetic resonance imaging (MRI) determined patients with lumbar disc herniation or IDD.

IDD patients were classified according to Pfirrmann Grading System (22) including 10 cases in grade III, 5 cases in grade IV, and 5 cases in grade V. Normal NP tissues were obtained from patients in the Department of Spinal Surgery, Affiliated Hospital of Jining Medical College. A total of 5 control patients with traumatic spine (2 tissue samples in C5/6 and C6/7 segments from patients with cervical fractures; 1 in L1/L2 segment from patient with L1 fracture) and 2 females (1 tissue sample in C5/6 segment from patient with cervical fracture, and 1 in T11/12 segment from patient with T11 fracture) having an average age of 42.6 (range 30–50). Their medical histories revealed no presence of pre-existing disc degeneration, spinal disorders or previous spine-related surgeries. This information was further confirmed by both CT and MRI. Patients with infections, degenerative spinal stenosis, idiopathic scoliosis or previous lumbar disc surgery were excluded. No statistical differences were found in age and gender between experimental and control groups (both P>0.05). The NP tissues were dissected carefully during surgery under a stereotaxic microscope (SZ61/SZ51, Olympus, Japan), and later subjected to various analyses based on the following procedures.

### MiR-21 and PDCD4 expressions tested by qRT-PCR

NP tissues were lysed with lysis buffer (250 µL) and proteinase K (final concentration: 800 μg/mL) at 55°C until the liquid turned clear, and incubated 20 min at 70°C. RNA was extracted using 1 mL Trizol (Invitrogen, USA). Reverse transcription-polymerase chain reaction (RT-PCR) was performed using 5 µg RNA (miRNA cDNA Synthesis kit; TaKaRa Biotechnology Co., Ltd., China), with a reaction volume of 20 µL. Real time quantitative PCR (qRT-PCR) was done using 0.5 µL reverse transcription (qRT-PCR Detection kit; TaKaRa Biotechnology Co., Ltd.). Reaction reagents (20 µL) were as follows: 10 µL SYBR Premix Ex TaqTM II; 0.8 µL PCR Forward Primer (10 μM); 0.8 µL PCR Reverse Primer (10 μM); 0.4 µL ROX Reference Dye (50×); 2.0 µL cDNA template; 6.0 µL dH_2_O (sterile purified water). PCR amplification was performed under the following conditions: after pre-denaturing with 45 cycles of 10 s at 95°C, 95 s at 5°C, and 34 s at 60°C, melting curve analysis was performed. After the end of the reaction, PCR amplification curve and melting curve were estimated, and the standard curve was drawn. Normalization was conducted applying U6 snRNA, which was used as an internal reference. The relative value of miR-21 was expressed using 2^-ΔΔCT^ [ΔΔCT = (CT_miR-21_-CT_U6_) experimental group - (CT_miR-21_-CT_U6_) control group]. The primers used to amplify miR-21 and U6 fragments using Primer Premier 5.0 software (Premier, Canada) were: miR-21: RT: 5′-GTCGTATCCAGTGCAGGGTCCGAGGTATTCGCACTGGATACGACTCAACA-3′; forward primer: 5′-GTGCAGGGTCCGAGGT-3′, reverse primer: 5′-GCCGCTAGCTTATCAGACTGATGT-3′; U6: RT: 5′-AACGCTTCACGAATTTGCGT-3′; forward primer: 5′-CTCGCTTCGGCAGCACA-3′, reverse primer: 5′-AACGCTTCACGAATTTGCGT-3′. The SYBR Prime Script mRNA qRT-PCR kit from TaKaRa was used to detect the expression of *PDCD4* via PCR amplification.

### Culture of NP cells

The NP tissue was separated under aseptic condition, cut into pieces, and digested with PBS containing 0.25% trypsin (Gibco-BRL, USA) for 40 min. The liquid was removed, and NP cells were washed with PBS and further digested with PBS and 0.025% type II collagenase (Invitrogen) for 4 h. After filtration and centrifugation at 500 *g* for 5 min, the supernatant was removed. NP cells were seeded into culture dishes in complete culture medium [DMEM/F12 supplemented with 15% fetal bovine serum (FBS, Gibco-BRL), 1% streptomycin/penicillin], and incubated in 5% CO_2_ (v/v) at 37°C, for 3 weeks. The medium was changed twice a week. The developed NP cells (passage number = 0–1) were used for subsequent experiments.

### Luciferase analyses

Cells growing well and sound were seeded onto a 6-well dish with a density of 1.0×10^6^ cells per well, added with Opti-MEM (Gibco), and transfected after cells were 90% confluent. Then, 15 µL (50 μM) miR-21 mimics and corresponding PDCD4 luciferase reporter gene vector (50 ng; mutant and wild-type PDCD4 plasmids, Shanghai GenePharma Co., Ltd., China) were added to Opti-MEM (100 µL); lipoetamine 2000 diluted in Opti-MEM (100 µL) was added to the mixture of miR-21 mimics and corresponding PDCD4 luciferase reporter gene vector; each well was added with 800 µL serum-free medium, and with the miR-21/PDCD4 mixture; cells were incubated for 6 h in a CO_2_-incubator, replaced with a new medium, and then collected after transfection (48 h). Fluorescence activity was detected using Dual-luciferase assay kit (E2920, Promega, USA).

### Cells transfection

The blank control group, negative control group (transfected with miR-21 negative sequences), miR-21 inhibitor group (transfected with miR-21 inhibitors), miR-21 mimic group (transfected with miR-21 mimics) and PDCD4 siRNA group (transfected with PDCD4 siRNAs) were established. The day before transfection, cells were seeded into 6-well dishes, and then 2 ml of medium was added to each well. Cell density had to be around 50–60% when transfecting. The medium used was then discarded, and cells washed twice with Opti-MEM I medium. Opti-MEM (11.5 mL) was added to each well. Opti-MEM I medium (250 µL) was utilized to dilute 5 µL miR-21 inhibitor, miR-21 mimics (Shanghai GenePharma Co, Ltd.), corresponding negative controls, and PDCD4 siRNAs (Shanghai GenePharma Co, Ltd.). Cells were developed for 5 min at room temperature until the final concentration of 50 nM was reached. Lipofectamine 2000 (Invitrogen) was diluted and mixed carefully with the above diluted transfections, and cultured 20 min at room temperature. Then, the above mixture was added into each well containing cells and medium (500 µL/well) and mixed equally; the dish was incubated in 5% CO_2_-incubator at 37°C, and after 6 h, medium was replaced with a fresh DMEM (Biowest, France) medium containing 10% FBS. Cells were collected after 48–72 h of transfection.

### Cell growth tested using cell counting Kit-8 (CCK-8)

The medium was renewed with 100 µL/well (96-well); then, 10 µL CCK-8 were added into each well (Research Institute of Tongren Chemistry, Japan), and the blank control group was set (with medium only). Both groups were developed for 1 h at 37°C. Medium was transferred to Eppendorf Tubes^¯^, and absorbance was evaluated at 24, 48, and 72 h. Zero was set as the value for the blank control group. The absorbance of each well at 450 nm was recorded on a microplate reader, and cell proliferation was estimated using pre-defined absorbance values. In each group, the average value of 3 wells was obtained, and the proliferation curve was drawn; the experiment was conducted 3 times.

### PDCD4, MMP-2 and MMP-9 mRNA expressions

A cDNA template was developed with a mRNA cDNA kit (Takara, Japan), and PCR amplification was conducted using SYBR Prime Script mRNA qRT-PCR kit (Takara). The reaction conditions were: 95°C for 30 s, 95°C for 5 s, 60°C for 30 s, for 40 cycles. Relative quantification (RQ) of target genes were calculated applying the 2^-ΔΔCT^ method. PCR primers were synthesized by Shanghai Sangon Biological Engineering Technology Co., Ltd., China (Table 1).

**Table:**
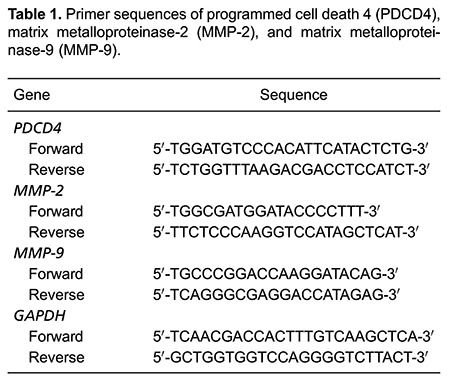

### Western blot

Total protein was extracted from each transfection group, and samples containing target protein were separated by SDS-PAGE electrophoresis; *in situ* electrophoresis was transferred to poly(vinylidene fluoride) PVDF membranes, which were sealed in sealing fluid. The primary antibody (PDCD4, 1:1000; c-Jun, 1:800; p-c-Jun, 1:800, Abeam, USA) was added, and membranes were shaken, and then cultured overnight at 4°C. Membranes were washed 3 times for 10 min using rabbit antibody 1×TBST (1:5000, Jackson, USA) or rat antibody (1:10000, Jackson), as a second antibody. Enhanced Chemiluminescence (ECL, Nanjing KeyGen Biotech. Inc. China) detection reagent was prepared and added to the membranes, which were allowed to react for 3–5 min. The detection liquid was drained and membranes were scanned in a darkroom. Protein bands were analyzed using Quantity One software (BIO-RAD, USA), and protein RQ is reported as the ratio of protein absorbance to the internal-reference absorbance.

### Statistical analysis

Data were analyzed using SPSS 18.0 software (SPSS Inc., USA), and results are reported as means±SD. Differences between 3 or more groups were evaluated by analysis of variance, and differences between two groups were tested using *t*-test. The correlation of miR-21 expression and Pfirrmann grades was estimated using Spearman's correlation analysis. P<0.05 indicated statistically significant differences.

## Results

### MicroRNA-21 was overexpressed in IDD patients

**Figure 1:**
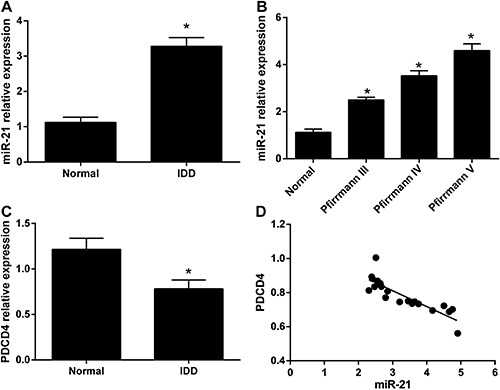
*A*, Expression of microRNA-21 (miR-21) from patients with intervertebral disc degeneration (IDD) and from normal controls. *B*, miR-21 expression from healthy controls and patients with Pfirrmann III, IV, V; *C*, programmed cell death 4 mRNA (PDCD4) expression in healthy controls and IDD patients; *D*, correlation analysis of miR-21 expression and Pfirrmann grades. *P<0.05, compared with normal controls (Student *t*-test).

As shown in Figure 1A, B, C, D, the miR-21 expression in IDD patients was significantly higher than that in healthy controls (P<0.05). The miR-21 expression was progressively higher in patients with Pfirrmann III, IV, V (P<0.05). In patients with IDD, the expression of PDCD4 was evidently decreased compared with that of healthy controls (0.782±0.096 *vs* 1.215±0.123, P*<*0.05). Correlation analysis showed that miR-21 expression was positively correlated with Pfirrmann grades (r=0.920, P<0.001), but negatively correlated with expression of PDCD4 (r=-0.857, P<0.001).

### PDCD4 was a target gene of miR-21

**Figure 2:**
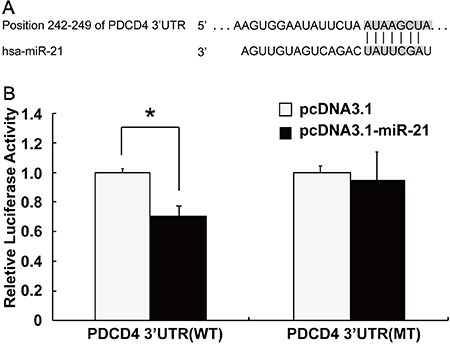
MicroRNA-21 (miR-21) directly targeting programmed cell death 4 (PDCD4). *A*: combined sequences of PDCD4 mRNA and microRNA-21 in 3′-UTR binding site; *B*, Dual-luciferase reporter analysis results: the activity of luciferase showed no significant difference in the group transfected with carriers of miR-21 and mutant (MT) PDCD4 mRNA (P>0.05), while the activity of luciferase was significantly decreased in the group transfected with carriers of miR-21 and wild type (WT) PDCD4 mRNA expressions (P<0.01); *P*<*0.05, pcDNA3.1-miR-21 group (transfected) compared to pcDNA3.1 group (negative control) (Student *t-*test).

The combined sequences of PDCD4 mRNA and miR-21 in the 3′-UTR binding site is demonstrated in Figure 2A. The result of dual-luciferase reporter analysis showed that, after transmitting the carrier containing the luciferase gene to 3′UTR binding site of *PDCD4*, the increased miR-21 inhibited the activity of luciferase. After mutation of the 3′UTR binding site of *PDCD4* and miR-21, activity of luciferase was not inhibited (Figure 2B).

### MiR-21 expression promoted cell growth

**Figure 3:**
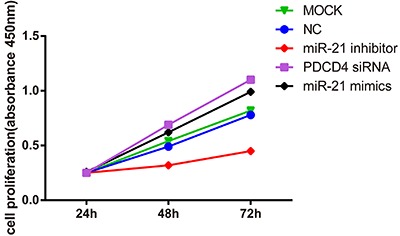
Growth curve of cells in each group after transfection (MOCK: blank control group; NC: negative control group; PDCD4: programmed cell death 4; miR-21: microRNA-21). Cell proliferation was promoted in PDCD4 siRNA group and miR-21 mimics group; thus, the inhibition of microRNA-21 could remarkably suppress cell proliferation.

Cell growth in the miR-21 inhibitor group was obviously inhibited when compared to blank control and negative control groups (both P<0.05); cell growth in PDCD4 siRNA and miR-21 mimics groups were higher than that in miR-21 inhibitor, blank control and negative control groups (all P<0.05). While not significant, differences in cell growth were found between blank control and negative control groups (P>0.05). No significant difference in cell growth was detected between PDCD4 siRNA and miR-21 mimics groups (P>0.05). The results demonstrated that miR-21 could promote cells proliferation. *PDCD4*, the target gene of miR-21, could inhibit cell proliferation (Figure 3).

### PDCD4, MMP-2 and MMP-9 mRNA expressions after transfection with miR-21

**Figure 4:**
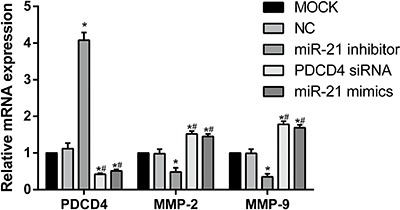
Programmed cell death 4 (PDCD4), matrix metalloproteinase-2 (MMP-2) and matrix metalloproteinase-9 (MMP-9) mRNA expressions after transfection (MOCK: blank control group; NC: negative control group; miR-21: microRNA-21). In the PDCD4 siRNA and miR-21 mimics groups, the expression of PDCD4 mRNA was lower compared with MMP-2 and MMP-9 mRNA (P<0.05). After miR-21 was inhibited, the expression of PDCD4 mRNA was significantly increased, but MMP-2 and MMP-9 mRNA were decreased. *P<0.05, compared with MOCK and NC groups; ^#^P<0.05 compared with miR-21 inhibitor group (Student *t-*test).

PDCD4 mRNA expression in the miR-21 inhibitor group was significantly higher than the other four groups (all P<0.05). PDCD4 mRNA expressions in PDCD4 siRNA and miR-21 mimics groups were significantly lower when compared to those in the blank control and negative control group (all P<0.05). MMP-2 and MMP-9 mRNA expressions were obviously higher in PDCD4 siRNA and miR-21 mimics groups than those in miR-21 inhibitor, blank control, and negative control groups (all P<0.05); furthermore, MMP-2 and MMP-9 mRNA expressions in miR-21 inhibitor group were significantly lower as compared with those in blank control and negative control groups (all P<0.05). PDCD4, MMP-2 and MMP-9 mRNA expressions were not significantly different between blank control and negative control group, as well as between PDCD4 siRNA and miR-21 mimics groups (all P>0.05). Our results revealed that miR-21 inhibited PDCD4 mRNA expression. Moreover, miR-21 also promoted expressions of MMP-2 and MMP-9 mRNA. On the contrary, PDCD4, the target gene of miR-21, inhibited expressions of MMP-2 and MMP-9 mRNA (Figure 4).

### PDCD4, c-Jun, p-c-Jun expressions after transfection with miR-21

**Figure 5:**
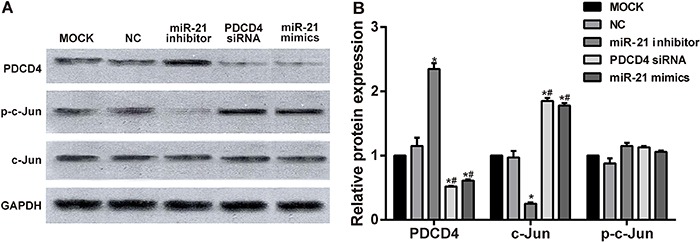
Programmed cell death 4 (PDCD4), c-Jun and p-c-Jun expressions after transfection. *A*, Western blot analysis of each group; *B*, comparison of PDCD4, c-Jun, and p-c-Jun expressions in each group. The expression of PDCD4 was decreased in both PDCD4 siRNA group and miR-21 mimics group, but the expression of c-Jun was elevated in both groups. The inhibition of miR-21 led to an increase of PDCD4 expression and decrease of c-Jun expression, while no differences in p-c-Jun expressions were detected among the five groups. *P<0.05, compared with blank control (MOCK) and negative control (NC) groups; ^#^P<0.05 compared with miR-21 inhibitor group. Comparisons among multiple groups were conducted using one-way ANOVA and pairwise comparisons were conducted using *t-*test.

PDCD4 protein expressions in miR-21 inhibitor group were strongly higher than those in PDCD4 siRNA, miR-21 mimics, blank control, and negative control groups (all P<0.05). Moreover, PDCD4 expressions in PDCD4 siRNA and miR-21 mimics groups were clearly lower than those in blank control and negative control groups (all P<0.05). P-c-Jun expressions in PDCD4 siRNA and miR-21 mimics groups were higher than those in miR-21 inhibitor, blank control, and negative control groups (all P<0.05). Besides, p-c-Jun protein expression in miR-21 inhibitor group was significantly lower than that in blank control and negative control groups (all P<0.05). PDCD4 and p-c-Jun expressions was not significantly different in blank control and negative control groups, as well as in PDCD4 siRNA and miR-21 mimics groups (all P>0.05). Meanwhile, c-Jun protein expression was not significantly different in miR-21 inhibitor, PDCD4 siRNA, miR-21 mimics, blank control and negative control group (all P>0.05). These results indicated that miR-21 inhibit PDCD4 protein expression, and also proved that PDCD4 was the target gene of miR-21. MiR-21 promoted p-c-Jun protein expression, and the target gene of miR-21, PDCD4, inhibited p-c-Jun protein expression (Figure 5).

## Discussion

Previous studies have suggested that miRNAs may play an indispensable role in regulating cell development, metabolism, and apoptosis of degenerated cartilage (14,15). However, the potential roles of miRNAs in the pathogenesis of IDD remain largely uncharacterized. In this regard, this study aimed to investigate the role of miR-21 in the pathogenesis of IDD. The results of our study demonstrate that miR-21 was strongly expressed in IDD patients, and positively correlated with Pfirrmann grades, suggesting that miR-21 could play a crucial role in IDD. Furthermore, miR-21 could promote the proliferation of NP cells by targeting PDCD4 and its downstream signaling molecules including c-Jun and MMPs.

Previous research reveals that elevated levels of miR-21 might be caused by local inflammation, and are correlated with post-trauma reactions in IVD. Furthermore, increased NP cells proliferation caused by up-regulation of miR-21 may be a potential mechanism in IDD development (1). A recent study has demonstrated that miR-21 promotes neoplastic cell transformation by repressing tumor suppressor genes including RECK, PTEN, TPM1 and PDCD4 (23). PDCD4, a tumor suppressor gene, is involved in cell apoptosis, transformation, invasion, as well as tumor progression (24). PDCD4 could influence different cellular translation levels as well as transcription pathways in different tumor entities. On the translational level, carbonic anhydrase II (CAII) is down-regulated in PDCD4 over-expressing cells (25). Moreover, PDCD4 could interfere, at the transcriptional level, in the specificity protein 1 (Sp1)/Sp3 of the urokinase receptor promoter motifs, by phosphorylation of the Sp transcription factors in colorectal cells (26). Asangani et al. (27) suggested that the 3′-UTR region of the PDCD4 mRNA is a target of the miR-21; high miR-21 concentrations caused a down-regulation of PDCD4, as well as an induction of intravasation, invasion and metastasis of tumor cells. Our finding is consistent with earlier studies, and confirms the role of miR-21 in the regulation of PDCD4 in NP cells.

Our research also found that miR-21 could promote the proliferation of NP cells by targeting PDCD4 and its downstream signaling molecules including c-Jun and MMPs. Numerous studies demonstrated that MMPs, including MMP-2 and MMP-9, were positively associated with IDD grades (28–30). Furthermore, c-Jun was reported to be activated in disc cells and cell clusters in herniated disc tissues (31). Investigations have revealed that PDCD4 could inhibit c-Jun activation, as well as activating transcription factor-1 (AP-1)-dependent transcription through the downstream MAP4K1/JNK/AP-1 signaling pathway, in colon carcinoma cells (32,33). AP-1 could regulate multiple biological events, such as MMPs expressions and cell motility. The Jun protein family comprises c-Jun, JunB and JunD (34–36). The most significant enzymes in extracellular matrix remodeling are disintegrin and metalloproteinase with thrombospondin motifs and MMPs families, both of which are specialized in the degradation of extracellular matrix (37). It is well documented that MMPs, including MMP-2, MMP-9 and MMP-13, play important roles in the regulation of extracellular matrix degradation, a major cause in the pathology of IDD, mainly through regulating cell inflammation, extracellular matrix deposition and tissue rearrangement (38). Therefore, based on our results, it is reasonable to suggest that miR-21 may regulate the expression of MMP-2 and MMP-9 via PDCD4 targeting. In a study by Zhu et al. (34), miR-21 regulated PDCD4 expression at the post-transcriptional, as well as translational levels in HepG2 cells, increased phosphorylation of c-Jun protein and activated AP-1-dependent transcription of MMPs.

In conclusion, miR-21 could promote the proliferation of human degenerated NP cells by regulating PDCD4 expressions, increasing phosphorylation of c-Jun protein, and activating AP-1-dependent transcription of MMP-2 and MMP-9, suggesting that miR-21 may be a crucial biomarker in the pathogenesis of IDD. Due to the difficulties in obtaining the nucleus pulposus tissues, the sample size in the current study was rather limited. Therefore, further studies with a larger sample size should be conducted to validate the results of our study.
